# A research education program model to prepare a highly qualified workforce in biomedical and health-related research and increase diversity

**DOI:** 10.1186/1472-6920-14-202

**Published:** 2014-09-24

**Authors:** Elahé T Crockett

**Affiliations:** Department of Medicine, College of Human Medicine, Michigan State University, East Lansing, MI USA

**Keywords:** Innovative on-line biomedical research training, Mobile biomedical lab, Innovative lab tool-kit, Microscopy training, Health-care research training program, Team mentoring, On-line medical research education

## Abstract

**Background:**

The National Institutes of Health has recognized a compelling need to train highly qualified individuals and promote diversity in the biomedical/clinical sciences research workforce. In response, we have developed a research-training program known as REPID (Research Education Program to Increase Diversity among Health Researchers) to prepare students/learners to pursue research careers in these fields and address the lack of diversity and health disparities. By inclusion of students/learners from minority and diverse backgrounds, the REPID program aims to provide a research training and enrichment experience through team mentoring to inspire students/learners to pursue research careers in biomedical and health-related fields.

**Methods:**

Students/learners are recruited from the University campus from a diverse population of undergraduates, graduates, health professionals, and lifelong learners. Our recruits first enroll into an innovative on-line introductory course in Basics and Methods in Biomedical Research that uses a laboratory Tool-Kit (a lab in a box called the My Dr. ET Lab Tool-Kit) to receive the standard basics of research education, e.g., research skills, and lab techniques. The students/learners will also learn about the responsible conduct of research, research concept/design, data recording/analysis, and scientific writing/presentation. The course is followed by a 12-week hands-on research experience during the summer. The students/learners also attend workshops and seminars/conferences. The students/learners receive scholarship to cover stipends, research related expenses, and to attend a scientific conference.

**Results:**

The scholarship allows the students/learners to gain knowledge and seize opportunities in biomedical and health-related careers. This is an ongoing program, and during the first three years of the program, fifty-one (51) students/learners have been recruited. Thirty-six (36) have completed their research training, and eighty percent (80%) of them have continued their research experiences beyond the program. The combination of carefully providing standard basics of research education and mentorship has been successful and instrumental for training these students/learners and their success in finding biomedical/health-related jobs and/or pursuing graduate/medical studies. All experiences have been positive and highly promoted.

**Conclusions:**

This approach has the potential to train a highly qualified workforce, change lives, enhance biomedical research, and by extension, improve national health-care.

## Background

Little attention has been given to the lack of diversity and related disparities, which exist in health-related research and clinical practices in the United States. Large gaps still remain in knowledge and in practice of science fields, and racial/ethnic minorities continue to have higher rates in diseases, disabilities and premature deaths than non-minorities [1, 2]. For example, while breast cancer has a higher incidence in white women, the mortality rate from breast cancer is higher in black women [2]. Additionally, the biomedical research workforce does not mirror the diversity of U.S. For example, a recent study indicates that the proportion of women and Black faculty in science departments of medical schools is lower than the proportion in similarly research-intensive university science Departments [3]. Further, there is a low percentage of tenured faculty versus the tenure-track pool for minorities, specifically with regards to African American women in science departments of medical schools [3]. Recently, the National Institutes of Health (NIH) has identified a set of 10-year health objectives with two overarching goals, to increase the quality and years of healthy life, and to eliminate health disparities [4]. Subsequently, the NIH has recognized a compelling need to promote greater diversity in the biomedical, behavioral, social and clinical sciences research workforce [4, 5]. In response to the NIH’s recommendation to prepare a trained workforce with researchers from diverse backgrounds, the College of Human Medicine at Michigan State University (MSU) has developed a research education program (REPID: Research Education Program to Increase Diversity in Health Researchers) to address the need of our nation for qualified researchers, eliminate the lack of diversity and thus to tackle existing problems of health disparities. By focusing on the inclusion of individuals from underrepresented, minority, and diverse backgrounds in research education, the REPID program (http://www.repid.msu.edu) has created a supportive environment for the training of motivated individuals from underrepresented, minority, and diverse backgrounds. This enables our trainees (throughout this document, the term “trainee” is used broadly to represent undergraduate, graduate, medical health professional students and lifelong learners supported by this training) to pursue careers in biomedical/health-related research to address the Nation’s biomedical and health-related research needs. The REPID program has also incorporated the best practices for training and sustaining the trainees in biomedical/health-related research through the design of the program as well as through partnerships with other existing programs at MSU (i.e., DREW, SROP, BEACON, ABLE, AGEP, please see abbreviations for complete names). Our program is helping to transform the academic culture and provide trainees from underrepresented, minority, and diverse backgrounds with the support they need to develop and sustain research careers in biomedical/health-related disciplines. Further, the REPID program has specifically assembled a strong group of mentors with nationally recognized research expertise in the focus areas of the National Heart, Lung, and Blood Institute (NHLBI); cardiovascular, pulmonary and blood disciplines. Because the funding has been provided through the NHLBI, research topics are limited to NHLBI focused areas. However, this program can be adapted for any areas of research program and development. Despite this, the combination of carefully providing standard basics of research education as well as research mentorship for the trainees has been successful and instrumental in fulfilling the goals of the REPID program. This program is ongoing and facilitates training of individuals with diverse backgrounds to obtain and enjoy successful and rewarding scientific/medical careers and thus improve the health care of the nation.

The goal of the REPID Program is to provide trainees with an enrichment experience that includes building confidence through comprehensive research training and experiences designed to inspire passion in trainees to pursue research careers in cardiovascular, pulmonary, and hematologic disciplines. The program’s intention is to support undergraduate, graduate, medical/health professional, and lifelong students/learners from underrepresented, minority, and diverse backgrounds at the university to challenge the existing problems of limited diversity and health disparities in biomedical/clinical research and clinical practice. Further, the program contributes to the development of a highly qualified workforce in biomedical and health-related research for the improvement of health and health care. This support is both through academic and research experience as well as financial aid. Each trainee receives a scholarship that provides a stipend and housing allowance for the summer months, during which s/he is engaged in a hands-on research experience. The trainee also receives assistance to attend an academic conference as well as for materials necessary to complete the course and research work. Through an inspiring and supportive environment, the ultimate goal is to foster career development, accomplishments and advancement to enable participating individuals to become more competitive as researchers and to develop their skills as critical, well-trained scientists. This program is ongoing, and the program’s components, its operation and the past three years operation outcomes are presented and discussed in this article.

## Methods

### Recruitment of trainees/application process

**Figure 1:**
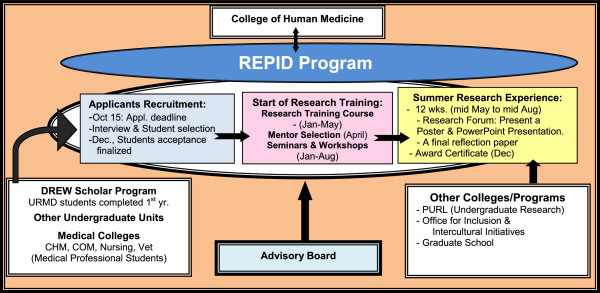
**Organizational and program chart.** This flowchart outlines the organizational and program structure. The program has three [3] steps: 1) Recruitment of the applicants, 2) Research training of basics and methods in biomedical research, and 3) followed by a hands-on-research experience. The overall functions and performance of the program is reviewed and advised by the advisory board of the program.

Individuals that participated in this research education program are from the University, voluntarily participate, and are not subject to experimental/clinical testing. Their rights are protected according to the University policies/regulations for the World Medical Association Declaration of Helsinki (http://www.wma.net/en/30publications/10policies/b3/index.html), reviewed and approved by the REPID advisory board and NIH (R25HL108864-00). All trainees were recruited from the Michigan State University campus from a diverse population of students. The program is advertised through various mediums across campus, including the deans of colleges, and directors of minority organizations. Once a prospective trainee is familiar with the program they are able to submit an e-application that is available via the REPID website (http://www.repid.msu.edu) by the October 15th deadline. The applicants are required to submit their transcripts and a personal essay that details their intentions for applying to the program and why and how they are able to fulfill the REPID program’s mission. Following the application submission, 25–30 prospective trainees are selected for a personal interview. Each applicant is scored and ranked as discussed in the Results/Discussion section. An advisory board then selects 15–19 trainees to recommend for the program. The potential trainees are notified by November 30th and have four days to accept or refuse program admission. The advisory board is consisted of eleven members (11), all of whom are senior faculty and/or administrators at the University. Figure 1 outlines the organizational and program structure.

### Biomedical research training: basics and methods in biomedical research

This course is an innovative on-line University course that is specifically designed for the research training of trainees at all levels (undergraduates, graduates, health professionals, and lifelong learners). This course is not exclusive to REPID trainees; other students are able to register for this course. It covers research concepts and strategies, literature search, ethics, responsible conduct of research, authorships, safety regulations, methods and laboratory techniques applied in biomedical research. Examples of the methods and techniques include buffer solutions/reagents preparation, pH measurement and adjustment, sterility technique and cell culture, slide preparation and use of a microscope, how to record research observations, data collection/ analysis, and presentation of research data in scientific formats (abstract, article, poster, and oral presentation). To learn and master the laboratory technical skills, trainees will incorporate the use of a research laboratory Tool-Kit (a lab in a box; My Dr. E.T. Lab Tool-Kit), as well as a digital microscope kit. Each trainee has his/her own laboratory Tool-Kit to learn and practice various lab techniques/skills as well as on-line instruction (a live interactive course with the instructor using the Adobe Connect system). Lectures/PowerPoints are uploaded on-line while each class is video-recorded and available to trainees for their own learning purposes.

### Workshops, research forums, and conferences

Trainees participate in two workshops, which include guest speakers, mentors, graduate/medical students and students from other research programs. These workshops and seminars are designed to enhance trainee’s learning experiences with communication, networking, and other professional skills. During these sessions the trainees have the opportunity to discuss and share their research experiences with others, i.e. peer coaching. The selected workshops focus on time and stress management, study skills, social issues related to race and cultural diversity and health care.

### Mentor selection hands-on summer research experience

Mentors are carefully selected from the University faculty and recruited based on the type of research they do and how compatible their field of study is with the program’s research interests, which focus mainly on pulmonary, cardiovascular and hematological disciplines. All mentors are well recognized for their national/international reputations (as judged by their extramural funding), have a passion in research, and an outstanding record of student training and mentorship. Each trainee identifies his/her research interest(s) and future career goal(s), which allows placement with a mentor within the same category. Prior to matching research trainees with mentors, a couple of months prior to the start of summer research training, the trainees are provided with the names/websites of all potential mentors to review, they will then select their top three choices for mentors. Before meeting with their mentor, each trainee is required to review his/her mentor’s website, perform a literature search of the mentor’s research work, become familiar with the mentor’s field of study/practice, write a 2–3 page review article about the mentor’s research work and present a PowerPoint-based presentation to her/his peers at the conclusion of the online course- this session is in-person. While this project is an assignment for trainees to practice and improve their skills in literature search, writing and oral presentations, it also strengthens the trainee’s confidence and ability to participate and work with his/her research mentor/team. Trainees participate in research for 10-12 weeks through hands-on research with their mentors.

## Results

**Figure 2:**
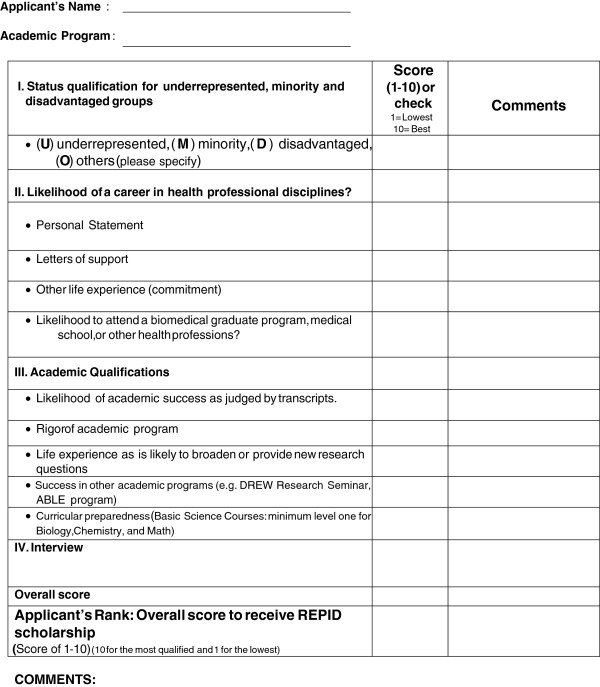
**Evaluation and grading form for REPID candidate.** This evaluation form was used to rank the candidates.

All of our trainees were recruited from the Michigan State University campus from a diverse population of undergraduates, graduates, health professionals, and life-long students/learners (Table 1 and 2 show demographic representations). Health professionals are individuals enrolled in medical school or pursuing a master’s degree in nursing, Epidemiology, public health or other health related programs (Table 2). About 25–30 applicants were selected for a personal interview, and each was scored and ranked using a grading system (Figure 2), and then based on their scores 15–18 trainees were recommended by an advisory board. Consistently, there was a larger population of female trainees with African American ethnicity background during the past three years of the training (i.e., Ratio 2:1 female:male). Once the trainees were admitted they were enrolled in the online hybrid research-training course.The research training program consists of two components: The first, and most important, includes teaching the standard of basics and methods in biomedical research, followed by a 12 week hands-on summer research experience (Figure 1). The foundation of the program introduces trainees to the standards of biomedical research concepts and methodology. This training assures that trainees gain the appropriate knowledge and information about what research is and how it is performed. They also acquire the basic technical skills necessary to function in a biomedical research laboratory or an epidemiology/clinical research team setting. This objective is accomplished through an on-line introductory course (i.e.; Basics and Methods in Biomedical Research), which is presented to the trainees prior to the summer hands-on research experience. The first and last classes are in-person, which allows the trainees to interact with each other and the instructor. Further, the trainees are given the opportunity to request in-person training for lab skills. This innovative approach allows trainees to have the satisfaction of learning on their own, while having the option to ask questions if they need help. This allows for trainees residing in remote parts of the State of Michigan or anywhere in the country to receive the research training. This research-training course significantly enhanced the trainee’s self-confidence in their ability to participate in the second part of the program. Through the on-line course, trainees also received assistance to create an individual development plans portfolio “IDPs” to record their research and academic goals, objectives, timelines, progress and accomplishments. The trainee identifies his/her short- and long- term academic/ research/ career goals and how s/he plans on achieving these goals with the program. The program’s mentor and teaching instructor/staff closely monitor each trainee’s research progress and the development of professional skills, while working with the trainee and providing instructions to overcome obstacles and barriers. Each trainee was required to submit weekly assignments, they were also required to perform lab tasks, submit a final paper, and present a Power Point presentation. These assignments allowed the instructor to monitor each trainee’s progress throughout the training program. After successful completion of the research course (to get a passing grade of 75% or more), the trainees then participated in the second part of the program, a 10–12 week summer hands-on research experience which allowed them to work with their selected research mentors.

**Table 1:** **Gender and minority demographic represented by REPID program**

	Asian*	Af. Am.	Mex. Am./ Chic.	His./Lat.	Am. Ind./Al. Native	White	UR	Dis. Bkg***
Female	4 (7%)	15 (30)	4 (7%)	7 (13%)	1 (2%)	2 (4%)	1 (2%)	6 (11%)
Male	3 (6%)	4 (7%)	3 (6%)	2 (4%)	0	4 (7%)	6 (11%)	3 (6%)
Total**	**7**	**19**	**7**	**9**	**1**	**6**	**7**	**9**
	**(13%)**	**(37%)**	**(13%)**	**(17%)**	**(2%)**	**(11%)**	**(13%)**	**(17%)**

*Abbreviations:* Ar. Am.: African American or Black; Mex. Am./Chic.: Mexican American/Chicano; His./Lat.: Hispanic/Latino; Am. Ind./Al. Native: American Indian/ Alaska Native; UR: Underrepresented; Dis. Bkg.: Disadvantaged Background.

*Asian (China, Korea, Burma, Vietnam).

**Some individuals qualify through several categories. The “total” shown for each category may be smaller or larger than the number of participants (i.e., fifty-one (51) trainees).

***Dis Bkg: Disadvantaged Background: Individuals who come from a family with an annual income below established low-income thresholds. These thresholds are based on family size, published by the U.S. Bureau of the Census. Individuals who come from a social, cultural, or educational environment such as that found in certain rural or inner-city environments that have demonstrably and recently directly inhibited the individual from obtaining the knowledge, skills, and abilities necessary to develop and participate in a research career.

**Table 2:** **REPID program undergraduate, graduate, health professional and gender demographic**

Class level	Undergraduate	Graduate/health professional*
2012	15 (F = 11, M = 4)	2 (F = 1, M = 1)
2013	16 (F = 12, M = 4)	2 (F = 1, M = 1)
2014	13 (F = 9, M = 5)	3 (F = 1, M = 2)
**Total**	**44 (84%)**	**7 (16%)**

*The health professionals as individuals enrolled in medical school or are pursuing a masters’ degree in nursing, epidemiology, public health or other health related programs. F, Female; and M, Male.

**Figure 3:**
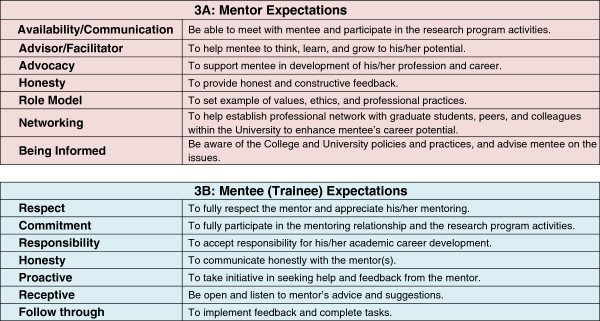
**Mentor and mentee (Trainee) expectations.**

**Figure 4:**
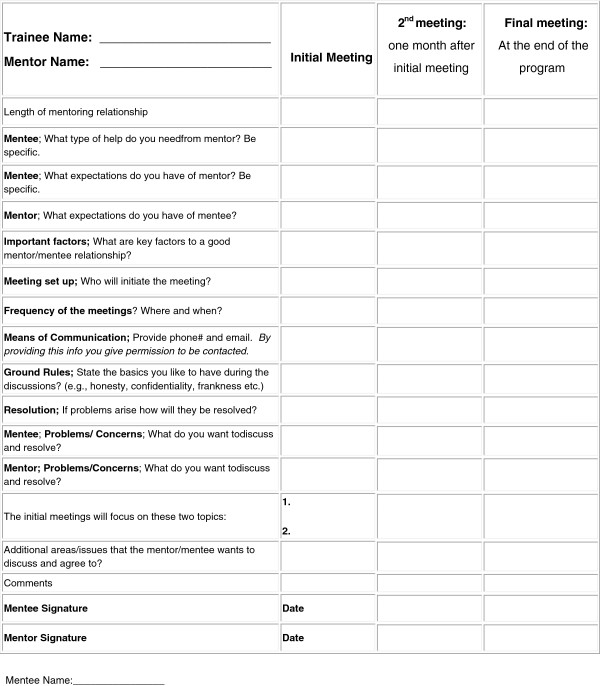
**Mentoring agreement form.** Mentee (Trainee/Student/Learner) - please process this form together with your primary research mentor- complete it before the start of your hands-on research project. The process facilitates communication between you and your mentor. All information between the mentor and the trainee remains confidential and is only shared with the director of the research program.

Prior to the start of the hands-on summer research experience, each trainee met with his/her mentor to complete a “Mentor-Mentee Expectations” application/agreement form and to review/discuss their research interest/projects (Figures 3 and 4 show mentor-mentee expectation and agreement forms). The goal was to assist, educate and assure the trainee of a good mentoring relationship as well as the goals and expectations for his/her summer research project. The guidelines suggested by Michigan State University, Center for Coaching & Mentoring, and other sources were adapted for the Mentor-Mentee Expectations as highlighted in Figures 3 and 4 Ref. [6–8]. Further, the trainees and mentors were provided with information and articles related to mentoring relationships [9]. During the initial mentor and mentee meeting, the program director was present and all the issues related to the program as well as mentor and mentee expectations were reviewed and discussed (Figures 3 and 4). The mentor and the mentee/trainee are encouraged to be pro-active in their relationship and should any problem arise that cannot be resolved then the program director is notified. The mentor-mentee agreement form (Figure 4) has a section about “Resolution;” which suggests means to consider if problems arise. Our program’s mentor-mentee matching has been successful so far, and among fifty-one (51) trainee- to- mentor matches we have had during the past three years of the program there was only one occasion when mentor mediation was necessary. This conflict was resolved by finding another mentor for the trainee. The success we have had so far clearly highlights the importance of finding and matching trainees with excellent mentors with a passion for training and nurturing trainees.

Trainees participated in research for 12 weeks through hands-on research with their research mentors. Throughout the 12-week period they also communicated with the program director (Program mentor) through e-mail, and had a minimum of three [3] face-to-face meetings with the program director to discuss their research experiences, IDPs portfolio and goals. Further, all of the trainees participated in a research forum scheduled for the end of July. Each trainee was required to present a poster and give an oral presentation about his/her summer research experience data and findings. This is a regional University research forum and over three hundred (300) students from across the country through other research programs, such as SROP, Summer Research Opportunity Program, also participated.

Upon completion of their research training, each trainee submitted a final written report about his/her summer hands-on research experience and how the training has fulfilled his/her expectations and the NHLBI- and REPID program’s goals and mission. In addition to the final report, both the trainee and the mentor fill out an exit survey, which gives insight to improve the program’s effectiveness. The program continues to mentor the trainees in their academic careers. Our trainees can update their progress personally, through the REPID website, or respond to inquiries sent out by the program staff. Our staff sends out inquires to past trainees 2–3 times per year.

### Program outcomes

During the first three years of the program, fifty-one (51) students were recruited to the program- thirty-six (36) trainees have completed their research program and the rest are in process of completion. The response and outcome of the research-training program has been outstanding. Early results are promising; of the thirty-six (36) trainees who have completed the REPID program, twenty-eight (28) have jobs or internships in scientific/health–related fields, or have been accepted into graduate/medical schools. Sixteen (16) trainees were offered continued, paid research positions in the host University laboratories/clinical departments, four [4] trainees were admitted into the College of Medicine, two [2] trainee were accepted into the University Dietetics Internship, two [2] trainees accepted positions with local hospitals, one [1] trainee accepted a position with the State of Texas as a Public Health & Prevention Specialist in HIV Research, one [1] trainee received an internship at the Children’s Environmental Health Network in Washington D.C., one [1] trainee accepted a position at a Biotech company, and three [3] trainees accepted positions in Health related areas (Table 3 shows details). Sixteen more trainees are currently completing their research training- the outcome of these trainees is not included in Table 3.

**Table 3:** **Post-program training outcome**

No. of Trainees* And%	Description of work ex-trainee is involved after completion of research training	Current position
16 (44%)	Offered continued research positions in research labs	Student
4 (11%)	Accepted into Medical School	Student
1 (3%)	Accepted into Physician Assistant Program	Student
2 (6%)	Accepted into Dietetics Internship and MS program- following completion one of the Ex-trainee was offered three different positions in Texas. Currently the ex-trainee working as Border Region Dietitian at H-E-B in Brownsville, TX	Dietician, Applying to PhD program
2 (6%)	Accepted into Master’s degree of Public Health MPH program	Grad Student
1 (3%)	Doing research and participating in medical residency program	D.O.
1 (3%)	Receive a faculty position as an instructor, and continuing her research work.	Faculty Instructor
1 (3%)	Accepted a research position in a biotech company	Production Chemist
1 (3%)	Received an Internship in Washington D.C. - Children’s Environmental Health Network	Student
2 (6%)	Accepted positions at local hospitals	Health care staff & scriber
3 (8%)	Accepted research related positions at university	Research Staff
1 (3%)	Accepted a research lab manger position. Planning to attend MD-PHD program	Research Lab Manager
1(3%)	Accepted a position as Public Health & Prevention Specialist in HIV Research for the State of Texas in Austin, TX	Health Specialist
10 (27%)	Attended national scientific conferences to present their research data/findings.	Students

Ex-Trainees’ status after completion of the research program. *A total of thirty-six (36) trainees have completed their research training. The number in parenthesis represents the percentage of the total 36 trainees.

## Discussion

The REPID research-training program was developed to recruit trainees from underrepresented, minority, and diverse backgrounds to pursue health related research careers with a focus on cardiovascular, pulmonary and hematologic fields. Information about the program and the recruitment/acceptance process are distributed to potential trainees during the summer and early fall terms. Early notification facilitates the candidate’s awareness of the program so they are able to plan their academic schedule. The selection and registration of trainees is accomplished through the review of their applications followed by an interview process (Figure 1 shows the program’s structure and process). The objective of this process is to make sure the prospective trainees are not only interested in biomedical research but that they also have the necessary science pre-requisite courses (i.e.; biology, chemistry, writing and math) for participating in research training.

The introductory on-line course that provides the basics and methods in biomedical research begins with the start of Spring Term (January 15). We chose the Spring Term to better prepare the trainees for their hands-on summer research experience. A fresh memory of research information and technical skills allow for a smooth transition and engagement in the challenges of their summer research projects. Further, the trainees, generally, have more free time during the summer, which allows them to fully engage in a hands-on research project. Our trainees have expressed enthusiasm about the on-line research-training course and the knowledge gained through the introductory course appears to be integral to their successful engagement with the hands-on research experience. Trainees have expressed feeling more confident and ready to get involved in their hands-on research projects because of the knowledge and skills gained during the introductory course. Knowing what to expect and how to engage in the research project greatly benefitted the trainees. Prior knowledge of what was required as a part of a research team and how to potentially coauthor a research publication are also valuable tools, being familiar with their mentors’ research was a central key to the engagement and successful completion of the research training. More importantly, general technical laboratory skills for working in a biomedical lab facilitated the trainee’s immediate participation in an on-going research project. Generally, trainees were happy with the use of the Lab Tool-Kit and Microscope. Skills learned for accurate pipetting, reagent and buffer preparation, cell culturing techniques and microscope use made trainees comfortable participating in biomedical research projects concerning cell isolation and tissue culturing, histology, and morphometric analysis.

One of the strengths of this research-training program is the emphasis on the direct interaction of the trainee with his/her mentor(s) and graduate students throughout training. A good mentor relationship is central to implementation of a successful training program. High-quality mentoring and career guidance for the trainees is essential and must be recognized. For this reason we have placed great emphasis upon the selection of mentors and judiciously matching mentors with trainees. It should be noted that each trainee is different and mentoring must be customized, adjusted, and directed to meet the trainee’s needs. Creation of the IDPs portfolio to record research and academic goals, objectives, timelines, progress and accomplishments is another important aspect for implementation of a successful mentorship. This is a learning tool for the trainee to identify and achieve professional development needs and career objectives by designing, monitoring, and determining his/her progress and success. Additionally, it facilitates communication between trainees and their mentors. It also promotes a clear definition of the expectations of each trainee from the outset of the training experience. Further, writing the IDPs portfolio and reviewing it with the mentor will assure quality mentoring and career guidance throughout the training period. The trainees are encouraged to continue this practice for their future goals and career plans. The program director served as an additional mentor to the trainees. Her caring and friendly mentoring relationship with trainees is believed to have played an important role in the success of this program. Further, the trainees greatly enjoy the informal gatherings and workshops, which have had a positive and educational impact, bringing the trainees closer to each other, which promoted sharing, interactions and networking.

Numerous studies and reports have clearly identified a lack of diversity in the health-related workforce and biomedical institutions. A study done by Leboy and Madden examining limitations of diversity in basic science workforces succinctly identified four major areas that could have positive influences and set trends to improve diversity. Our REPID program’s goal is aligned with noted areas; “early and continuing efforts to diversify the pipeline by increasing numbers of women and minorities getting advanced degrees;” and, “introducing mentoring programs and coping strategies to help women and minorities.” This study also indicates that although women and minorities are provided with the opportunities to advance, they are often unable to move forward and participate in higher positions within institutions. While this study does present limitations (less than 2% of all institutions were examined), it highlights an important issue. We must continuously work together toward common goals, which recognize the essential need for high-quality mentoring and career guidance for the next generation of researchers and educators. We believe the REPID program is on the right track. Educating individuals from underrepresented, minority, and diverse backgrounds and providing them with knowledge and experience allows them to compete within the field of biomedical science and healthcare as well-qualified, highly competitive members of the workforce with the ability to achieve the highest ranks of academia and public health institutions.

In summary, a combination of on-line research methods teaching and a summer hands-on research experience has been instrumental for the successful training of sixteen (16) to nineteen (19) trainees from diverse and minority backgrounds each year. The response and outcome of the research-training program has been great. Trainees confidently enjoyed positive experiences with their mentors and peers and the program has provided them with new and exciting opportunities while bringing them closer to their career goals. Trainees have stated that REPID education and training played a key role in their professional development- one trainee wrote:

“Most labs want to see previous experience…*REPID gave me the doorway to walk through*”.

## Conclusions

This innovative program provides research training and education to trainees from diverse and minority backgrounds, which fosters active participation in biomedical and health-related research. The program helps facilitate integration of confident, highly trained professionals from diverse underrepresented backgrounds within the biomedical and health-related workforce. These new health research professionals will assure a highly competent workforce and challenge some of the problems associated with diversity and health disparities in our society and around the world.

## Authors’ information

EC is an associate professor of medicine and experimental pathology at the College of Human Medicine. She serves as the director of the REPID research education program. She is a research scientist with expertise in the area of leukocyte biology and inflammation/reperfusion injury, and her research projects have been funded through American Heart Association and NIH. She also teaches medical students in the areas of general pathology and immunopathology. Furthermore, she has developed medical education programs including the Masters of Science in Surgery, and an integrated MD-PhD program. Moreover, she is an authentic representational artist.

## Abbreviations

ABLE: Advanced baccalaureate learning experience at MSU
AGEP: Alliances for graduate education and the professorate at MSU
BEACON: Center for the study of evolution in action at MSU
DREW: Charles Drew science scholars program at MSU- provides academic and social support for high achieving students pursuing science and math degrees
IDP: Individual development plans portfolio
MSU: Michigan State University
NHLBI: National Heart, Lung, and Blood Institute
NIH: National Institutes of Health
NIAID: National Institute of Allergy and Infectious Diseases
REPID: Research education program to increase diversity among health researcher
SROP: Summer Research Opportunity Program
URMD: Under-represented minorities and disadvantaged backgrounds.
